# Flow injection analysis of water. Part 1: Automatic preconcentration determination of sulphate, ammonia and iron(II)/iron(III)

**DOI:** 10.1155/S1463924693000185

**Published:** 1993

**Authors:** J. S. Cosano, M. D. Luque de Castro, M. Valcárcel

**Affiliations:** Department of Analytical Chemistry Faculty of Sciences University of Córdoba Córdoba E-14004 Spain

## Abstract

This paper describes a simple flow-injection (FI) manifold for the determination of a variety of species in industrial water. The chemical systems involved in the determination of ammonia (formation of Indophenol Blue), sulfate (precipitation with Ba(II)), and iron (complexation with 1,10-phenanthroline with the help of a prior redox reaction for speciation) were selected so that a common manifold could be used for the sequential determination of batches of each analyte. A microcolumn of a suitable ion exchange material was used for on-line preconcentration of each analyte prior to injection; linear ranges for the determination of the analytes at the ng/ml levels were obtained with good reproducibility. The manifold and methods are ready for full automation.


Journal of Automatic Chemistry, Vol. 15, No. 4 (July-August 1993), pp. 141-146

### Flow injection analysis of water. Part 1: Automatic preconcentration determination of sulphate, ammonia and iron(II)/iron(III)

**J. S. Cosano, M. D. Luque de Castro and M. Valcárcel**

*Department of Analytical Chemistry, Faculty of Sciences, University of Córdoba, E-14004 Córdoba, Spain*

*This paper describes a simple flow-injection (FI) manifold for the determination of a variety of species in industrial water. The chemical systems involved in the determination of ammonia (formation of Indophenol Blue),, sulfate (precipitation with Ba(II)), and iron (complexation with 1,10-phenanthroline with the help of a prior redox reaction for speciation) were selected so that a common manifold could be used for the sequential determination of batches of each analyte. A microcolumn of a suitable ion exchange material was used for on-line preconcentration of each analyte prior to injection; linear ranges for the determination of the analytes at the ng/ml levels were obtained with good reproducibility. The manifold and methods are ready for full automation.*

### Introduction

Flow injection analysis (FIA) is becoming a valuable tool for routine analysis due to its proven features for developing sensitive, reproducible and rapid determinations [1, 2]. Demand for routine analysis is particularly high in the industrial field and the authors' research team has as one of its goals the establishment of methods to solve general or specific problems in industry. A recent challenge for the team was to develop methods for the determination of low concentrations of sulphate, ammonia and Fe(II)/Fe(III). The first two analytes had to be determined at low levels because the permitted concentration in the surrounding industrial environment is at the ng/ml level. The speciation of iron as Fe(II)/Fe(III) was required to control the concentration of both oxidized states of iron, which is important in power plants due to their erosive action on the surface of turbine blades.

The FI manifold to be designed for this task had to be a single, versatile manifold which could be used for large batches of each analyte, in such a way that after establishing the working conditions of one method the determinations could be performed automatically (unattended).

Initially, individual methods for the analytes were developed, taking into account the later integration of all three. The second stage is the integration and automation of the system, including the development of a computer program which will control the different automatic units of the FI manifold as well as data acquisition and treatment.

### Experimental

### *Instruments and apparatus*

A Jenway 6100 spectrophotometer connected to a Knauer x-t recorder and furnished with a Hellma 178.012 QS flow-cell (18 µl inner volume) was used. A Gilson Minipuls-3 eight-channel programmable peristaltic pump, two Rheodyne 5041 manual injections valves (one of them acting as selecting valve) and a laboratory-built dual injection system with inner coupled valves were also used.

### *Reagents*

All chemicals used were analytical reagent grade.

#### *Reagents for iron speciation*

Aqueous solutions of 0.1% (w/v), 1,10-phenanthroline, 0.5 M acetic acid/sodium acetate buffer of pH 4.6, 0.15 M $H_2SO_4$, 0.1 M EDTA+0.1% (w/v) $CuSO_4$, and 1 g/l standard solutions of Fe(II) [from $(NH_2Fe(SO_4)_2$ in 0.18 M $H_2SO_4$] and Fe(III) [from $Fe(NO_3)_3 \cdot 9H_2O$ in 1 N $HNO_3$] were used. A redox column of copperized cadmium and a chelatant resin of iminodiacetic acid (50–100 mesh) packed in a 5 cm × 2 mm column were also used.

#### *Reagents for sulphate determination*

Aqueous solutions of 5% (w/v) $BaCl_2 \cdot 2H_2O$ + *0.05%* polyvinyl alcohol, 0.01 M HCl, 0.3 M NaCl, 1 g/l of standard of sulphate (from $K_2SO_4$) and Bio-Rad AG1-X8, 100–200 mesh anionic resin packed in a 15 cm × 2 mm column were used.

#### *Reagents for ammonia determination*

Aqueous solutions of 24 g/l sodium hypochlorite + 0.24 g/l of sodium nitroprusside, 60 g/l of phenol + 10% (v/v) ethanol adjusted to pH 12.4 with NaOH solution, 0.1 M NaCl, 1 g/l standard solution of ammonia from $(NH_4)_2SO_4$, and Amberlite CG-120 cationic resin packed in a 15 cm × 2 mm column were used in this determination.

0142-0453/93 $10.00 © 1993 Taylor & Francis Ltd.

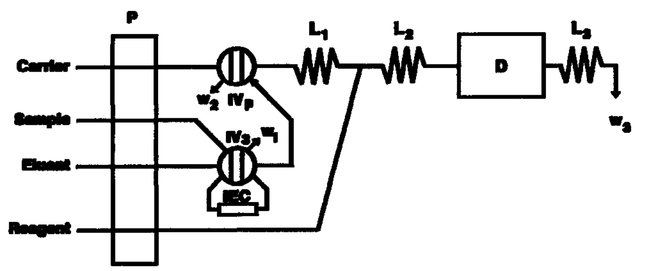

*Figure 1. Manifold for the determination of ammonium or sulphate.*

### Results and discussion

Optimization was a problem—the usual method could not be used because the final aim was the integration of the three methods using a single manifold. Thus the optimization study searched for compromises between values of variables of the three systems, in order to design a final manifold which could be as simple as possible, with minimal sacrifice of the optimum working conditions of each method. The three methods are described in the order in which they were developed. First, the most complex was optimized and then the other two were adapted to the restrictions imposed by the first. The chemical systems for the three analytes were selected after a review of current FIA literature and preliminary experimentation.

### *Method for the determination of ammonium ion*

The *chemical* system selected for the determination of ammonium ion was the reaction between hypochlorite ion and phenol in the presence of the analyte in a basic medium to yield a coloured product with maximum absorption at 636 nm (Berthelot reaction [3]). Photometric determination of ammonia using a pH indicator [4–8] requires a gas-diffusion unit to separate the analyte from the matrix, which was not compatible with the future integration; and also the preparation of the reagents for development of the Nessler reaction is more laborious [8] than that of the selected method.

The method for ammonium ion was first developed as the complexity of the chemical system involved is slightly higher than that of the other two.

The *manifold* designed for this method is shown in figure 1. In this FI system the sample was circulated through the loop of valve $IV_3$ (load position), in which an ion exchange column was located thus preconcentrating the analyte. After a preset preconcentration time, $IV_3$ was switched to the injection position and an NaCl stream eluted the analyte from the resin to fill the loop of the injection valve $IV_p$, which injected its contents into the phenol basic solution acting as a carrier. After formation of a chloramine between phenol and ammonium along reactor $L_1$, the reactant plug merges with the hypochlorite solution, and the blue product was formed along reactor $L_2$ and monitored at 636 nm in its passage through the flow-cell.

The *optimization* of the variables affecting the system was marked by the relatively slow kinetics of the derivatizing reaction, despite the use of a catalyst (nitroprusside) in the hypochlorite solution. Although the use of temperatures above room values increased the reaction rate, room temperature was selected to avoid the use of a thermostatic bath which could complicate the system. So, a relatively long length of reactors $L_1$ and $L_2$ (300 and 400 cm, respectively) and the presence of an auxiliary reactor at the outlet of the detector, to avoid bubble formation, were required. The length of the preconcentration column was 15 cm; the maximum length which did not cause overpressure drawback in the FI system.

The FIA peak increased by increasing the preconcentration time, but above 150 s the increase of sensitivity was almost nil—possibly owing to saturation of the ion exchange material packed into the column. Table 1 summarizes the optimum values of the FIA and chemical variables for this determination.

### *Features of the method*

A series of solutions with varying concentration of standard ammonium ion were used under the working conditions listed in table 1 to establish the linear range of the calibration curve, which was located between 325 and 1400 ng/ml if preconcentration of the analyte was performed. The preconcentration factor, calculated as the ratio between the determination limit of the calibration curve without and with preconcentration was 2.7. Two linear ranges (betweeen 0.88–25 μg/ml and between 25–70 μg/ml) were obtained by applying the method without the preconcentration step.

The reproducibility of the method, with and without the preconcentration step, was calculated by using 11 different samples in triplicate injection and it provided an r.s.d. of 2.55 and 0.89%, respectively (see table 3).

### *Method for the determination of sulphate*

Several chemical systems all with photometric detection, were checked before selecting a turbidimetric method using $BaCl_2$ as derivatizing reagent. The use of Ba-dimethylsulphonazo(III) [DMSA(III)] to yield a displacement reaction of the analyte with monitoring at 656 nm of the released DMSA(III), proposed in the FIA literature by several authors [9–13] did not give reproducible and sensitive results and the base-line was noisy. On the other hand, Thorin [14] (precipitation of sulphate with an excess of $Ba(ClO_4)_2$ in an organic medium) also produced poor, irreproducible and low sensitive results, even after assaying several surfactants in both aqueous and organic media.

The turbidimetric method (formation of $BaSO_4$ and monitoring at 480 nm) although not sufficiently sensitive [15–17], produced more reproducible results and was more appropriate for the FI manifold. Again, a preconcentration step allowed the concentration limit required to be obtained.

*Table 1. Optimum values of variables for the determination of ammonia.*

| | Variable | | Studied range | Optimum value |
|---|---|---|---|---|
| FIA variables | Flow rate | Total | 0.5–2.0 ml $min^{-1}$ | 1.4 ml $min^{-1}$ |
| | | Eluant | 0.5–2.0 ml $min^{-1}$ | 1.1 ml $min^{-1}$ |
| | Reactor length ($\phi_i$ = 0.5 mm)* | $L_1$ | 50–600 cm | 300 cm |
| | | $L_2$ | 50–600 cm | 400 cm |
| | | $L_3$ | — | 315 cm |
| | Injection volume | | 100–500 µl | 300 µl |
| Physical variables | Temperature | | 10–80 °C | 20 °C |
| Chemical variables | Carrier | Phenol | 10–50 $g \cdot l^{-1}$ | 50 $g \cdot l^{-1}$ |
| | | Ethanol | 0–33% | 10% |
| | | pH | 9.0–13.0 | 12.4 |
| | Reagent | Hypochlorite | 3.5–24 $g \cdot l^{-1}$ | 24 $g \cdot l^{-1}$ |
| | | Nitroprusside | 0.5–1.0 mM | 0.8 mM |
| | | pH | 12.0–13.0 | 12.4 |
| | Eluant | NaCl | 0.01–0.5 M | 0.3 M |
| Preconcentration variables | Column length | | — | 15 cm × 2 mm |
| | Preconcentration | | 60–210 s | 150 s |
| | Elution time | | 30–80 s | 65 s |

*$\phi_i$ = inner diameter.

*Table 2. Optimum values of variables for the determination of ammonia.*

| | Variable | | Studied range | Optimum value |
|---|---|---|---|---|
| FIA variables | Flow rate | Total | 1.0–4.0 ml $min^{-1}$ | 3.2 ml $min^{-1}$ |
| | | Eluant | 1.0–3.0 ml $min^{-1}$ | 2.5 ml $min^{-1}$ |
| | Reactor length ($\phi_i$ = 0.5 mm)* | $L_1$ | 50–600 cm | 300 cm |
| | | $L_2$ | 50–600 cm | 400 cm |
| | | $L_3$ | — | 315 cm |
| | Injection volume | | 100–500 µl | 220 µl |
| Chemical variables | Carrier | Barium chloride | 1–10% | 5% |
| | | Polyvinyl alcohol | 0.001–0.2% | 0.05% |
| | Reagent | Hydrochloric acid | 0.0–0.3 M | 0.01 M |
| | Eluant | NaCl | 0.01–0.5 M | 0.1 M |
| Preconcentration variables | Column length | | — | 15 cm × 2 mm |
| | Preconcentration | | 60–600 s | 600 s |
| | Elution time | | 15–60 s | 26 s |

*$\phi_i$ = inner diameter.

In the flow injection manifold in figure 1, the sample was preconcentrated in the ion-exchange column placed in the loop of the secondary valve ($IV_3$). The analyte was then eluted by passage of the NaCl solution when the valve was switched to the injection position; the eluted analyte filled the loop of the primary valve ($IV_p$), which injected the sulphate solution into a carrier stream of $BaCl_2$ aqueous solution which contained a surfactant (polyvinyl alcohol) to minimize deposition off the precipitate on the walls through the FI system. The acid stream merging with the main channel was intended to dissolve the precipitates formed by other anions present in the sample matrix and also retained and preconcentrated in the microcolumn. The length of reactors $L_1$ and $L_2$ led to a drop of sensitivity.

The optimum values of variables (flow-rates, injection volume, preconcentration and elution times, type of resin and concentration of reagents) for developing this method are listed in table 2. A study of the preconcentration time allowed a relationship to be found between the analytical signal provided by the preconcentrated analyte (FIA peak) and the product of the preconcentration time × conconcentration of sulphate ion in the sample:

$$A = 0.016 \pm 0.007 + 1.28 \cdot 10^{-4} \pm 6 \cdot 10^{-6} \cdot [SO_4^{=}] \cdot T_p^{(*)}$$

$$r^2 = 0.98$$

$^{(*)}$A = absorbance, $[SO_4^{=}]$ in µg/ml,

$T_p$ = preconcentration time (s).

*Table 3. Features of the methods for the determination of ammonia, sulphate and Fe(II)/Fe(III).*

| Analyte | Method | | Value | Method | Condition | Value |
|---|---|---|---|---|---|---|
| $NH_4^+$ | I | A | $A = 0.151 \pm 0.002 + 0.0173 \pm 0.0002 \cdot [NH_4^+]$ | | | $A = 0.192 \pm 0.003 + 0.206 \pm 0.004 \cdot [NH_4^+]$ |
| | | B | 0.9995 | | | 0.998 |
| | | C | 0.880 – 25 | | | 0.325 – 1.4 |
| | | D | 0.89 (10 µg ml$^{-1}$) | | | 2.55 (0.4 µg ml$^{-1}$) |
| | II | A | $A = 0.37 \pm 0.01 + 0.0078 \pm 0.0002 \cdot [NH_4^+]$ | | | |
| | | B | 0.9992 | | | |
| | | C | 25–70 | | | |
| | | D | 0.61 (50 µg ml$^{-1}$) | | | |
| $SO_4^{=}$ | | A | $A = -0.0127 \pm 0.005 + 0.002302 \pm 9 \cdot 10^{-6} \cdot [SO_4^{=}]$ | I | $T_p = 90$ s | $A = -0.002 \pm 0.0153 \pm 0.0003 \cdot [SO_4^{=}]$ |
| | | B | 0.9999 | | | 0.999 |
| | | C | 10 – 80 | | | 0.822 – 10 |
| | | D | 1.50 (50 µg ml$^{-1}$) | | | 1.70 (5 µg ml$^{-1}$) |
| | | A | | II | $T_p = 180$ s | $A = -0.003 \pm 0.003 + 0.030 \pm 0.001 \cdot [SO_4^{=}]$ |
| | | B | | | | 0.996 |
| | | C | | | | 0.474 – 5.0 |
| | | D | | | | 1.68 (2 µg ml$^{-1}$) |
| | | A | | III | $T_p = 600$ s | $A = 0.007 \pm 0.001 + 0.093 \pm 0.001 \cdot [SO_4^{=}]$ |
| | | B | | | | 0.9997 |
| | | C | | | | 0.087 – 1.5 |
| | | D | | | | 1.56 (1 µg ml$^{-1}$) |
| Fe | I $Fe^{++}$ | A | $A = 0.016 \pm 0.005 + 0.0385 \pm 0.0007 \cdot [Fe^{++}]$ | I | $Fe^{++}$ $T_e = 50$ s | $A = 0.023 \pm 0.008 + 0.86 \pm 0.04 \cdot [Fe^{++}]$ |
| | | B | 0.9992 | | | ·0.996 |
| | | C | 1 – 9 | | | 0.030 – 0.300 |
| | | D | 1.70 (5 µg·ml$^{-1}$) | | | 1.85 (0.2 µg·ml$^{-1}$) |
| | II $Fe^{++}$ | A | $A = 0.006 \pm 0.006 + 0.038 \pm 0.001 \cdot [Fe^{++}]$ | II | $Fe^{++}$ $T_e = 35$ s | $A = 0.009 \pm 0.004 + 0.76 \pm 0.03 \cdot [Fe^{++}]$ |
| | | B | 0.999 | | | 0.996 |
| | | C | 1 – 9 | | | 0.050 – 0.400 |
| | | D | 1.75 (5 µg·ml$^{-1}$) | | | 1.88 (0.2 µg·ml$^{-1}$) |
| | III $Fe^{3+}$ | A | $A = -0.0028 \pm 0.0008 + 0.0274 \pm 0.0001 \cdot [Fe^{3+}]$ | III | $Fe^{3+}$ $T_e = 35$ s | $A = 0.002 \pm 0.002 + 0.49 \pm 0.01 \cdot [Fe^{3+}]$ |
| | | B | 0.99996 | | | 0.997 |
| | | C | 3 – 12 | | | 0.100 – 0.500 |
| | | D | 1.68 (8 µg·ml$^{-1}$) | | | 1.92 (0.3 µg·ml$^{-1}$) |

Notes

A: Equation. A = Abs, [] in µg·ml$^{-1}$

B: Regression coefficient ($r^2$)

C: Linear range (µg·ml$^{-1}$)

D: Relative standard deviation, % (concentration of the analyte)

$T_p$: Preconcentration time

$T_e$: Elution time.

### *Features of the method for the determination of sulphate*

A series of samples with different concentrations of sulphate were prepared for the calibration graphs; the linear range depended on the preconcentration time—see table 3. The reproducibility of the method was calculated for each preconcentration time by using 11 different samples (of 5, 2 and 1 µg/ml of sulphate for 90, 180, and 600 s of preconcentration time), which were injected in triplicate. When the method was applied without a preconcentration step the linear range was between 10–80 µg/ml.

### *Method for iron speciation*

The chemical system selected for developing this method was the orange complex formed between Fe(II) and 1,10-phenanthroline with maximal absorption at 510 nm. The Fe(III) was determined after reduction to Fe(II) by passage of the sample through a copperized-cadmium redox column. Thus, an aliquot of sample was used to determine Fe(II) and another aliquot was passed through the redox column to determine the total iron present in the sample: the concentration of Fe(III) being calculated and as the difference.

The flow injection manifold required for speciation of iron is shown in figure 2. The only difference between the manifold for the determination of ammonium ion and sulphate (figure 1) was a dual injection system [18, 19], one valve containing the redox column. The procedure for speciation requires two sample injections. When the three valves are in the filling position, the eluent fills the loops $V_1$ and $V_2$, bidistilled water fills the redox column (RC) and the sample passes through the chelatant resin (IEC). After the preconcentration interval, $IV_3$ is switched to the inject position, the $H_2SO_4$ solution elutes the analytes retained in the resin and the eluate fills first $V_2$ and then $V_1$. So the elution time, or interval, between the switching of $IV_3$ and the simultaneous switching of the inner coupled valves, depends on the species to be determined. When the dual valve is switched 50 s after $IV_3$, the volume of eluent containing the analytes fills $V_1$; it is sent to $L_1$ without passing through the redox column. Thus only Fe(II) is determined after merging with the reagent. However, when the elution time is 30 s the eluted

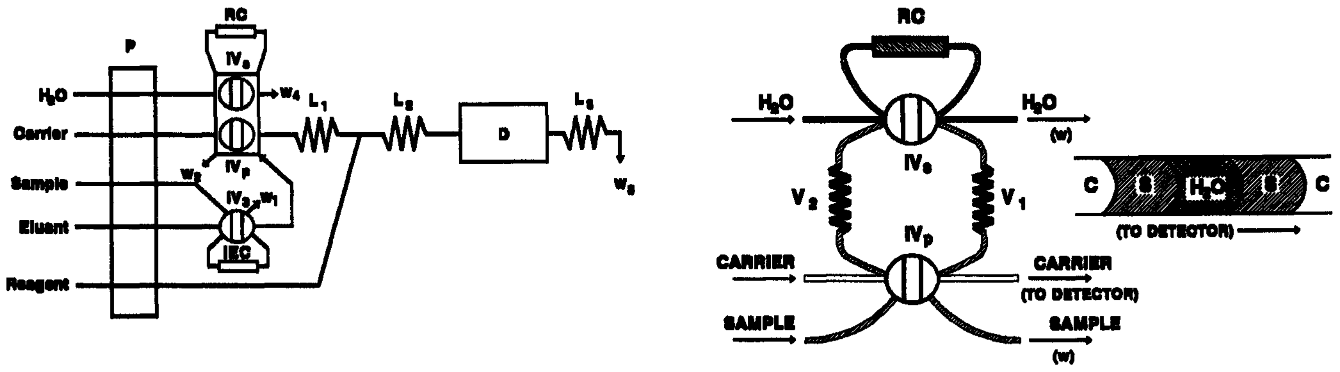

*Figure 2. Manifold for iron speciation (A) with innerly coupled valve system (B).*

*Table 4. Optimum values of variables for the speciation of iron.*

| Variable | | | Studied range | Optimum value |
|---|---|---|---|---|
| FIA variables | Flow rate | Total | 0.5–4.0 ml $min^{-1}$ | 1.4 ml $min^{-1}$ |
| | | Eluant | 0.5–4.0 ml $min^{-1}$ | 1.1 ml $min^{-1}$ |
| | Reactor length ($\phi_i$ = 0.5 mm)* | $L_1$ | 50–600 cm | 300 cm |
| | | $L_2$ | 50–600 cm | 400 cm |
| | | $L_3$ | — | 315 cm |
| | Injection volume | $V_1$ | 100–500 µl | 300 µl |
| | | $V_2$ | 100–500 µl | 220 µl |
| Chemical variables | Carrier | $AcOH/AcO^-$ buffer Conc. | 0.1–1.0 M | 0.5% |
| | | $AcOH/AcO^-$ buffer pH | 4.0–6.0 | 4.6 |
| | Reagent | 1,10-Phenanthroline | 0.04–1.0% | 0.1 M |
| | Eluant | $H_2SO_4^=$ | 0.01–0.5 M | 0.15 M |
| Preconcentration variables | Column length | | — | 5 cm × 2 mm |
| | Preconcentration time | | 120–300 s | 300 s |
| | Elution time | $T_{e1}$ | 15–40 s | 35 s |
| | | $T_{e2}$ | 40–60 s | 50 s |

*$\phi_i$ = inner diameter.

analytes fill $V_2$ and the simultaneous switching of $IV_p$ and $IV_s$ forces them to pass through the redox column; the Fe(III) present is reduced to Fe(II) before passing through $L_1$ and merging with the 1,10-phenanthroline solution. The FI peak obtained at the passage of the reactant plug through the detector corresponds to both the Fe(II) and Fe(III) eluted from the preconcentration column; so, the concentration of Fe(III) present in the original sample can be calculated as the difference between this signal and that obtained by injecting at a elution time of 50 s. A correction factor, taking into account the different dispersion degree undergone by each plug, must be applied.

The behaviour of such variables as flow rates, preconcentration and elution times, injection volumes, type of resin and reagent concentrations are listed in table 4.

### *Features of the speciation method*

The different dispersion at the detector of plugs for $V_1$ and $V_2$ meant that three calibration curves had to be run: two for Fe(II) at the two elution times (35 and 50 s), and one for Fe(III), reduced in RC at the elution time 35 s. Table 3 gives equations of the linear portion of the three calibration curves, the determination limit, and reproducibility for each analyte expressed as r.s.d. and calculated from 11 samples of 0.2 and 0.3 µg/ml of Fe(II) and Fe(III), respectively, injected in triplicate.

### Final remarks

The proposed methods were developed by using the single manifold depicted in figure 2. For the first two methods discussed (determination of ammonium and sulphate ions) valve $IV_s$ was not used. All three methods need the loop of the main injection valve to be changed, together with the flow-rate for the different channels. The solution for developing the analytical reaction and wavelength for monitoring, of course, also need to be altered. In this way the manifold is ready for the new step: automation for unattendant functioning of each method.

### Acknowledgement

Dirección General de Investigación Científica y Técnica is thanked for financial support for this research.

### References

1. Valcárcel, M. and Luque de Castro, M. D., *Flow Injection Analysis: Principles and Applications* (Ellis Horwood, Chichester, 1987).

2. Ruzicka, J. and Hansen, E. H., *Flow Injection Analysis* (John Wiley, New York, 1988).

3. Berthelot, M. E. P., *Report de Chemie Appliqué*, **284** (1859).

4. Van Son, M., Schothorst, R. C. and Den Boef, G., *Analytical Chimica Acta*, **153**, 271 (1983).

5. Nakata, R., Kawamura, T., Sakashita, H., and Nitta, A., *Analytical Chimica Acta*, **208**, 81 (1988).

6. Svesson, G. and Anfält, A., *Clinica Chimica Acta*, **119**, 7 (1982).

7. Clinch, R., Worsfold, P. J., and Sweeting, F., *Analytical Chimica Acta*, **214**, 401 (1988).

8. Schulze, G., Liu, C. Y., Brodowski, M., Elsholz, O., Frenzel, W., and Möller, J., *Analytica Chimica Acta*, **214**, 121 (1988).

9. Reijnders, H. F. R., van Staden, J. F., and Griepink, B., *Fresenius, Z. Anal. Chem.*, **295**, 410 (1979).

10. Kondo, O., Miyata, H., and Toei, A. *Analytica Chimica Acta*, **134**, 353 (1982).

11. Reijnders, H. F. R., van Staden, J. J., and Griepink, B., *Fresenius, Z. Anal. Chem.*, **295**, 122 (1979).

12. Ibid, **300**, 273 (1980).

13. Nakashima, S., Yagi, M., Zenki, M., Doi, M., and Toei, K., *Fresenius, Z. Anal. Chem.*, **317**, 29 (1984).

14. Persson, G. A., *Air and Water Pollution*, **10**, 845 (1966).

15. Krug, F. J., Bergamin, H., Zagatto, E. A. G., and Jorgensen, S., *Analyst*, **102**, **503** (1977).

16. van Staden, J. F., *Fresenius Z. Anal. Chem.*, **310**, 239 (1982).

17. Karlsson, M., Persson, J. A. and Möller, J., *Analytica Chimica Acta*, **244**, 109 (1991).

18. Ríos, A., Luque de Castro, M. D., and Valcárcel, M., *Analyst*, **110**, 227 (1985).

19. Ibid.

